# Response of Annual Herbaceous Plant Leaching and Decomposition to Periodic Submergence in Mega-Reservoirs: Changes in Litter Nutrients and Soil Properties for Restoration

**DOI:** 10.3390/biology10111141

**Published:** 2021-11-05

**Authors:** Xin Hu, Tingting Xie, Muhammad Arif, Dongdong Ding, Jiajia Li, Zhongxun Yuan, Changxiao Li

**Affiliations:** Key Laboratory of Eco-Environments in the Three Gorges Reservoir Region (Ministry of Education), Chongqing Key Laboratory of Plant Resource Conservation and Germplasm Innovation, College of Life Sciences, Southwest University, Chongqing 400715, China; sthuxin@email.swu.edu.cn (X.H.); xtt791141187@email.swu.edu.cn (T.X.); muhammadarif@swu.edu.cn (M.A.); dingdongdong@email.swu.edu.cn (D.D.); ljj133888@email.swu.edu.cn (J.L.); yuanzhongxun@email.swu.edu.cn (Z.Y.)

**Keywords:** Three Gorges Dam Reservoir, flooding stress, riparian zone restoration, annual plants, litter, buried sediment, soil nutrients

## Abstract

**Simple Summary:**

This research focuses on the leaching and decomposition of riparian zone plants, which lose mass and release nutrients due to changing water levels during their vigorous growth period. While different factors greatly influence litter decomposition, the change in soil characteristics over various depths and their relationship to litter are largely unknown in mega-reservoir settings. Current research explores how annual plants decompose and release nutrients while they are submerged in soggy circumstances. Flooding circumstances can hasten plant mass loss and nutrient release, as well as change soil and water characteristics. This research found that sediment hindered the loss of mass and C, N, and P elements while stimulating the release of the K element. The litter decomposition of annual herbaceous plants has minimal impact on the overall amount of carbon and nutrients in the soil when the soil is saturated with water. This is linked to water leaching and soil element transformation. However, this does not imply that the significance of litter for soil nutrition is minor. It is essential to investigate the continuing production of residual soil litter nutrients after the water level has receded.

**Abstract:**

Litter decomposition is an important soil nutrient source that promotes vegetation in deteriorated riparian zones worldwide. The periodic submergence and sediment burial effects on two prominent annual herbaceous plants (*Echinochloa crusgali* and *Bidens tripartite*) are little known in mega-reservoir settings. Our study focuses on the mass and carbon loss and nutrient release from *E. crusgali* and *B. tripartitle* litter and changes in soil properties, which are important for riparian zone rehabilitation in the Three Gorges Dam Reservoir, China. This study adopted the litter bag method to explore the nutrient change characteristics and changes in soil properties at different sediment burial depths under flooding scenarios. Three burial depths (0 cm, 5 cm, and 10 cm) were used for these two plants, and the experiment lasted for 180 days. The results revealed that the litter decay rate was high at first in the incubation experiment, and the nutrient loss rate followed the pattern of K > P > N > C. The relationship between % C remaining and % mass remaining was nearly 1:1, and the total amount of P exhibited a leaching–enrichment–release state in the decomposition process. Nutrients were changed significantly in the soil and overlying water at the first decomposition stage. Still, the total soil nutrient change was insignificant at the end, except for the 10 cm burial of *B. tripartitle*. Moreover, oxidation–reduction potential was the main factor in the litter decomposition process at different burial depths. This study indicated that sediment deposition reduced litter mass loss, slowed down the release of N and P, and retained more C, but promoted the release of K. Conclusively, in litter decomposition under waterlogging, the total soil nutrient content changed little. However, litter does more to the soil than that. Therefore, it is necessary to study the residual soil litter’s continuous output after the water level declines for restoration purposes.

## 1. Introduction

Litter decomposition is a fundamental component in any ecosystem cycle [1,2], which plays an important role in controlling nutrient transfer within and between aquatic and terrestrial environments [3]. Through decomposition and leaching, litter, as the main source and path of nutrient and organic matter in the soil, affects the nutrient exchange between plants and soil and can change the accumulation and loss of soil carbon [4,5]. Besides carbon, nutrient elements, such as N, P, K, Ca, and Mg, are released in litter decomposition. Substances released by litter decomposition can change soil properties and fertility and increase the available nutrients contained in the soil [5,6]. Extensive research has reported that litter decay’s impact factors generally cover resource quality and the external environment [7,8,9]. Resource quality includes the physicochemical properties of litter, such as the initial C, N, and P elements, and lignin and cellulose, which can determine the decay rate of litter [10,11]. The litter N plays an important prediction role in early decomposition, while C controls the decomposition in the form of lignin and cellulose in the late stage. In addition, microorganisms have a high demand for N and P nutrients. Temperature, moisture, and detritivores are examples of external environmental factors that affect decomposition directly or indirectly [12]. For example, temperature changes the mass loss and nutrient release rate of litter by affecting the activity of microbial decomposers [13,14]. As a result, changes in the environment may have a significant impact on litter mass and nutrient loss [12]. Previous research has shown that the initial litter decomposition rate is accelerated in underwater flooding conditions due to the rapid loss of soluble substances, such as K element, by flushing, leaching, and collecting aquatic fungi. At the same time, the degradation of stubborn matter in the later decomposition stage leads to a slow decomposition rate [15,16]. The decomposition at the later stage under flooding may be slower than that on land because the slumbering aerobic fungi under anoxic conditions cause litter decomposition to be reduced. However, some studies have speculated that decomposition is fast under flooding, possibly because aquatic fungi can degrade leaf litter more effectively than terrestrial fungi [15]. Many studies have focused on the decay of litter on the soil surface in forest or stream ecosystems, whether on land or underwater [10,11,12]. Little research has been conducted on annual herbaceous plants, which are characterized by dry–wet alternations [13].

Due to water fluctuation in the dry–wet area, organic matter and nutrient elements in the soil are removed, resulting in coarser soil substrate and reduced plant cover [17]. Nutrient elements would be released into the water through leaching [18]. Previous studies have shown that soil organic matter decomposition is inhibited by waterlogging conditions [19,20]. The soil contains more organic carbon due to the slow decomposition rate in the anaerobic water environment. The increase in organic carbon changes soil properties and causes changes in the activity of decomposing microorganisms, thus affecting litter decomposition. However, the relationship between the change in soil properties and litter decomposition under waterlogging conditions needs further study.

The Three Gorges Dam (TGD) on the upper reaches of the Yangtze River is the largest dam in China. Since the establishment of the Three Gorges Dam Reservoir (TGDR), a special water storage mode of “winter storage and summer drainage” has been followed, and the water level fluctuates, reaching a height of 30 m, in the TGDR [17,21,22]. As the ecotone between aquatic and terrestrial regions, the water-level-fluctuation zone is critical in controlling the exchange of nutrients and energy between adjacent habitats [3,23]. Due to the unseasonal environmental conditions in the water-level-fluctuation zone of the TGDR, many annual herbaceous plants in the region begin to die and decompose under the stress of submergence conditions during the vigorous growth period [24,25]. Annual herbaceous plants are always under flooding stress in the decomposition process, and the water body usually carries sediment during the impoundment [26]. However, sediment deposition would bury herbaceous litter at different depths, affecting the decomposition of litter and nutrient release to different degrees. In particular, *Poaceae*, represented by *Echinochloa crusgali*, and *Asteraceae*, represented by *Bidens tripartita*, have a large biomass and a broad reach inside the TGDR. In decomposition, the degradation and re-mineralization of litter have a greater impact on soil. Subsequently, soil can retain carbon and nutrients and become a growth source for plants during the next year [27,28,29]. Nevertheless, if many nutrients are enriched in the stream, it may become a potential factor for the occurrence of environmental problems such as phytoplankton blooms [30,31]. Therefore, it is important to study the nutrient release characteristics of annual herbaceous plants at different burial depths and their effects on soil properties under waterlogging conditions.

The construction of the TGDR has changed the ecological environment of several parts of the Yangtze River basin [21,22,25]. As water eutrophication causes environmental pollution, the potential factor may be the nutrient elements released by plant litter decomposition. Therefore, this research aims to determine nutrient loss characteristics from herbaceous plant leaching and decomposition under waterlogging conditions at various sediment burial depths, assess their effects on soil and overlying water properties, and study the relationship between litter and soil. We hypothesized that (i) sediment burial would inhibit litter mass and nutrient loss, and (ii) the slow release of litter nutrients would reduce soil nutrient enrichment. In this experiment, natural environmental conditions were reproduced as much as possible during the decomposition process.

## 2. Materials and Methods

### 2.1. Study Site and Experimental Materials

**Figure 1:**
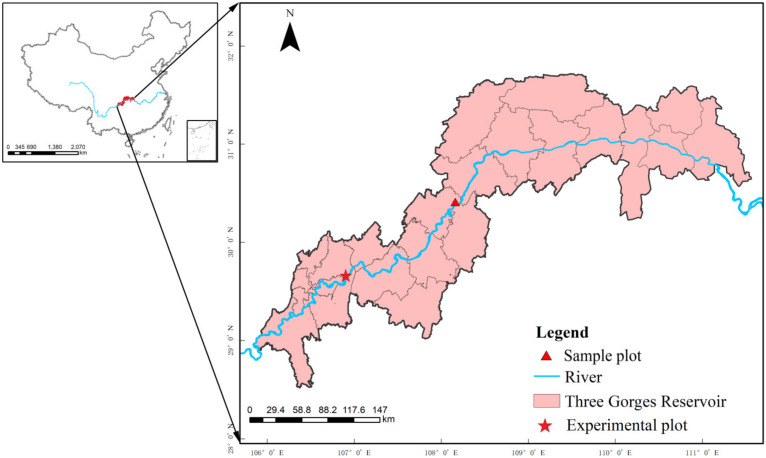
Locations of sample and experiment site in the riparian zone of the Three Gorges Dam, China.

The experimental materials were collected from the hydro-fluctuation belt of Shibao Town, Zhongxian County, Chongqing, China (30°36′ N, 108°06′ E) (Figure 1). The vegetation coverage in this area is mainly herbaceous. Among them, more plants are annual herbs, such as *Echinochloa crusgali* and *Bidens tripartita*, while perennial herbs account for a small proportion. The region has a mountainous climate, typical of the subtropical southeastern monsoon region, with plenty of sunshine and rainfall [24]. The annual average accumulated temperature is 18.2 °C, with 1327.5 h of sunshine. The average daily temperature of the coldest month is 8.5 °C, and the average daily temperature of the warmest month is 31 °C. The daily illumination rate is 29%, and the annual rainfall is up to 1200 mm. The most rain falls between June and August, with a relative humidity of 80% [22,23]. The soil in this region is classified as purple soil (Regosols in FAO Taxonomy or Entisols in USDA Taxonomy).

### 2.2. Experimental Design

*E. crusgali* and *B. tripartita* were dried to constant weight and put into litter bags (8 cm × 8 cm, diameter 0.5 mm × 0.5 mm) containing 3.0 g per sample bag. The experimental container was a box (with an open top) of 66 cm × 45 cm × 35 cm in dimensions. The experimental soil was mixed and placed in the box until the soil thickness reached 15 cm. The litter bags were placed on the soil’s surface and buried at depths of 0 cm, 5 cm, and 10 cm based on the sediment deposition characteristics of the water fluctuation zone in the TGDR. The experiment is designed as follows: 2 species (*E. crusgali* and *B. tripartita*) × 3 sampling times (30, 90, 180 d) × 3 repeats, a total of 18 boxes, each including three burial depths (0, 5, 10 cm). The sampling time was designed according to the actual flooding time of annual herbaceous plants. A total of 60 litter bags (30 for each species) were prepared, and the initial values of soil and plant samples were measured at the beginning of the experiment. After the experiment began, all the boxes were kept on the soil surface with 20 cm of water.

### 2.3. Treatments and Measuring Methods

The experiment began in October 2018. All treatments were carried out at the Ecological Research Base of the Key Laboratory of the Three Gorges Reservoir Region′s Eco-environment, Ministry of Education, Southwest University. Samples were collected from the treatment group on the 30th, 90th, and 180th days after the decomposition bags were placed in the light of the water-flooding rhythm of the hydro-fluctuation belt in the TGDR. After the bag was brought back to the laboratory, the soil impurities were slowly washed with tap water and then rinsed with ultra-pure water 3 times. The washed litter was put into the labeled envelope and put into the oven at 60 °C to dry to constant weight. The dry weight was weighed and then ground with a ball mill and passed through a 0.25 mm sieve to determine nutrient elements. The collected soil was air-dried to constant weight in the laboratory. After soil samples were ground, they were screened out with a soil sieve (2 mm, 1 mm, 0.25 mm) and marked to determine physicochemical properties (Table 1).

The total C and N contents in litter and soil were determined using an elemental analyzer (CHNS-O-VARIO EL Cude; Heraeus Elementar, Hanau, Germany). The total P and K contents in litter and soil were digested using a microwave digestion apparatus (SPEEDWAVE MWS-4) and determined using an inductively coupled plasma emission spectrometer (ICP-OES, Thermo Fisher Scientific ICAP 6300). The available phosphorus and available potassium contents in soil were determined using plasma emission spectrometry; ammonium nitrogen and nitrate nitrogen contents in soil and water were determined using an automatic discontinuous chemical analyzer (ClerverChem). The total nitrogen and total phosphorus contents in water were determined using a potassium persulfate digestion–UV spectrophotometer. The soil temperature was measured using a thermometer, soil pH value was measured using an acidometer, and the soil redox potential was determined using an oxidation–reduction potentiometer (oxidation–reduction potential, ORP).

### 2.4. Statistical Processing and Analysis

According to the mass remaining and the nutrient accumulation index (NAI) formula, the calculation is as follows:

Mass remaining = M_t_/M_0_ × 100%, (1)

NAI = (M_t_·X_t_)/(M_0_·X_0_) × 100% (2)

In the formula, NAI is the nutrient accumulation index, M_0_ is the initial dry weight (g) of the sample, M_t_ is the dry weight (g) of the sample at t, X_0_ is the initial concentration of the sample elements, and X_t_ is the concentration of the sample elements at t.

The data were analyzed using SPSS 22 (IBM, Chicago, IL, USA). Repeated measures ANOVA was used to analyze the effects of species, burial depth, time, and their interaction on nutrient elements. One-way ANOVA was used to analyze the changes in mass and different nutrient elements in two annual herbaceous plants with the same decomposition time and burial depth and to analyze the effects of different decomposition times on the nutrient element content in the soil around the litter and overlying water at the same burial depth. Duncan’s multiple range test was used to test the significance of the difference (α = 0.05). Linear regression was used to model the relationship between nutrients remaining and mass remaining in the litter. Principal component analysis (PCA) analyzed the correlation between nutrient elements and environmental variables in the litter decomposition process. Graphs were drawn using OriginPro 9.1 (Northampton, MA, USA).

## 3. Results

### 3.1. Dynamic Characteristics of Mass and Nutrient Elements in the Litter

**Figure 2:**
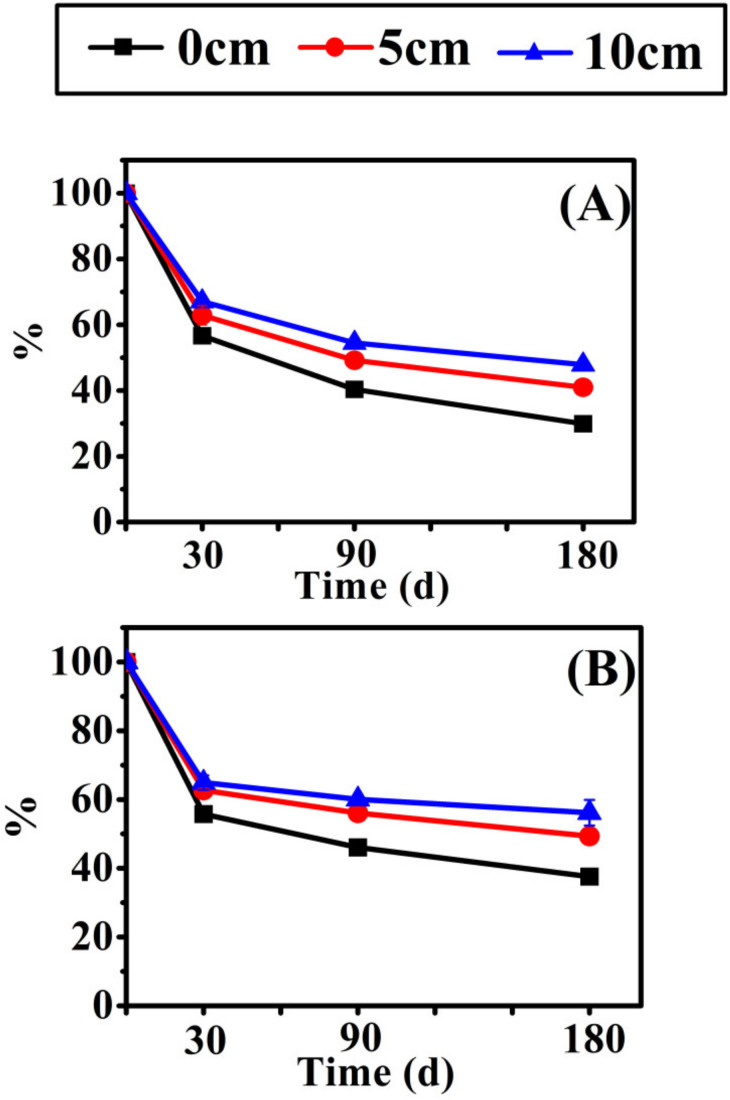
Percent mass remaining of *Echinochloa crusgali* (**A**) and *Bidens tripartita* (**B**) litter decomposition after 30, 90, 180 days at 0, 5, 10 cm burial depths under flooding. Mass remaining is expressed relative to the initial litter mass. Error bars indicate ± 1 S.D.

**Figure 3:**
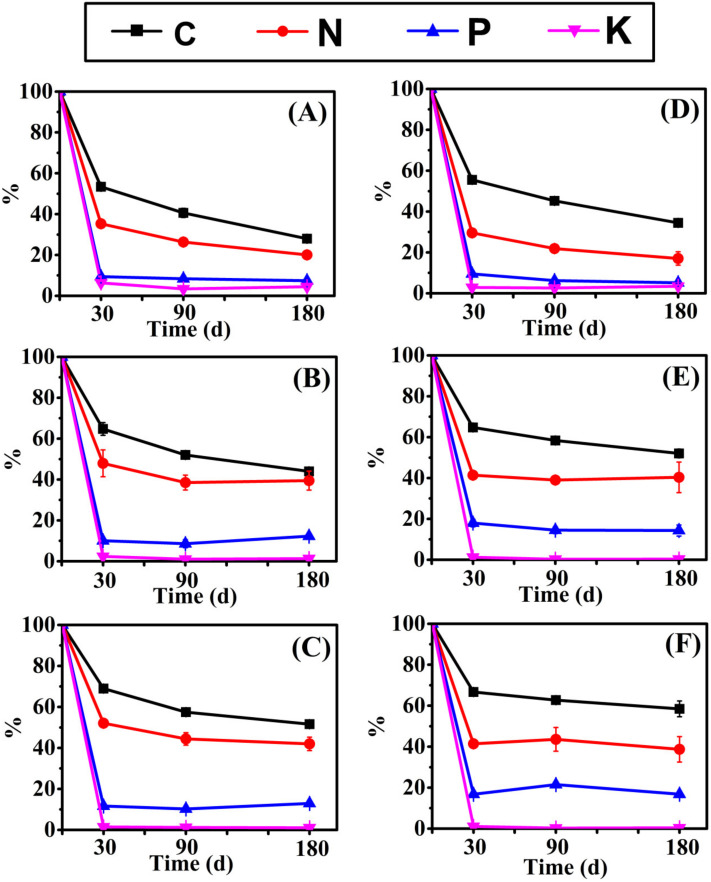
Carbon, nitrogen, phosphorus, and potassium NAI values of *E. crusgali* and *B. tripartita* litter decomposition after 30, 90, 180 days at 0, 5, 10 cm burial depths under flooding. (**A**) *E. crusgali* at 0 cm; (**B**) *E. crusgali* at 5 cm; (**C**) *E. crusgali* at 10 cm; (**D**) *B. tripartita* at 0 cm; (**E**) *B. tripartita* at 5 cm; (**F**) *B. tripartita* at 10 cm. NAI: nutrient accumulation index. NAI values are expressed relative to the initial litter mass. Error bars indicate ± 1 S.E.

**Figure 4:**
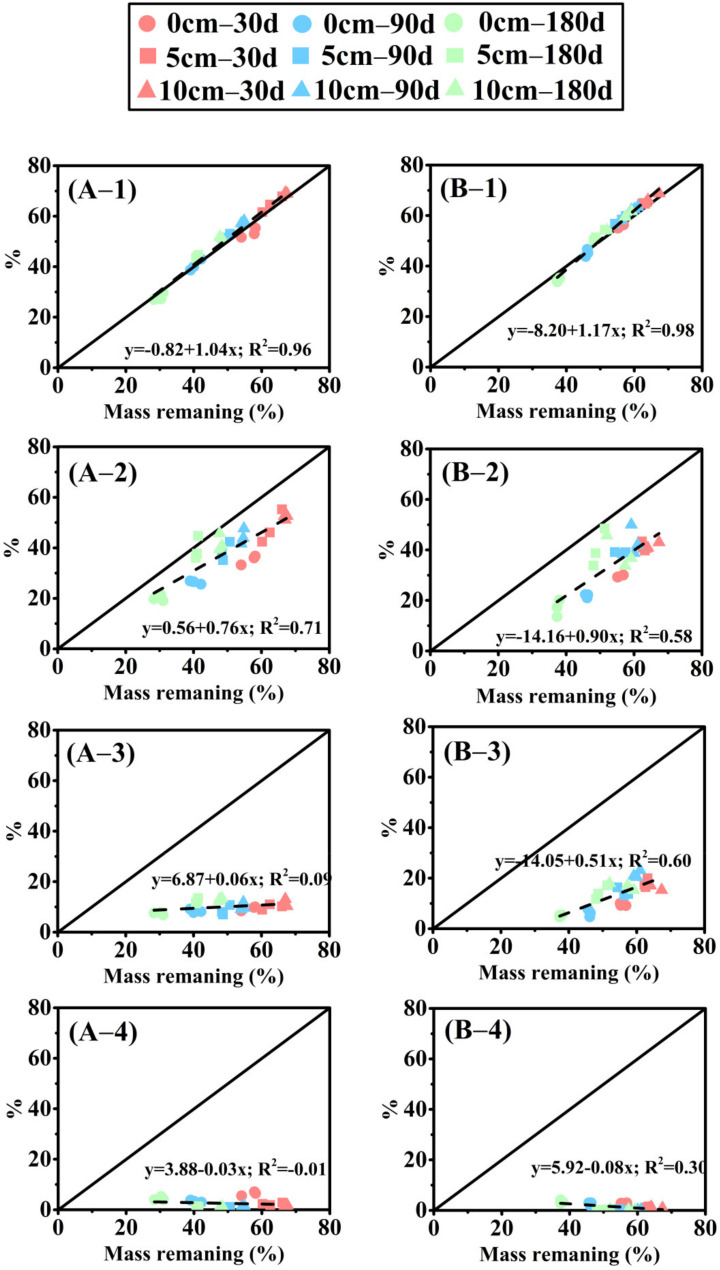
Relationship between percent litter mass remaining and percent C, N, P, and K mass remaining in *E. crusgali* and *B. tripartita*. (**A–1**) C in *E. crusgali*; (**A–2**) N in *E. crusgali*; (**A–3**) P in *E. crusgali*; (**A–4**) K in *E. crusgali*; (**B–1**) C in *B. tripartita*; (**B–2**) N in *B. tripartita*; (**B–3**) P in *B. tripartita*; (**B–4**) K in *B. tripartita*. Symbols represent sediment burial treatments, whereas colors represent collection dates. In each figure, regression lines are dashed, and the 1:1 line is solid.

The repeated measure variance analysis of the nutrient release of two herbaceous plants is shown in Table S1. Different species, burial depths of sediment, times, and interactions significantly affected the nutrient release of litter decomposition. The burial depth significantly affected the mass loss and element release from litter (*p* < 0.001). P and K changed little with time, and C and N content decreased continuously with decomposition. Remaining litter mass (Figure 2) and NAI nutrient accumulation index changes (Figure 3) point to burial depth for significant effects on litter. The decomposition at 10 cm depth was significantly slower than at 5 cm and 0 cm depth, and a deeper burial inhibited litter mass loss and nutrient release. In addition, the decomposition rate of *E. crusgali* was significantly faster than that of *B. tripartita* after 30 days of decay, depending on the curve slope. C, N, P, and K content decreased in the overall decomposition process of two annual herbaceous plants (*E. crusgali* and *B. tripartita*) at different burial depths. In contrast, the relationship between nutrient element changes did not depend on the burial depth. Among them, the release rate of K was the highest at 30 days; that is, the KNAI was the lowest among the four elements, the KNAI of each burial depth was less than 10% (*p* < 0.05), and the change was not obvious in the subsequent decomposition. The released amount of C in the litter was the smallest compared with the initial total amount. The litter loss rate of nutrients was K > P > N > C in the continuous decomposition process. The relationship between the % C remaining and the % mass remaining of the litter of two herbs was nearly 1:1 (*y* = −0.82 + 1.04*x*, Figure 4A–1; *y* = −8.20 + 1.17*x*, Figure 4B–1), while nutrient elements were less than 1:1 (Figure 4), indicating that the release rate of N, P, and K was always faster than that of mass. The fitting line of % N remaining was closer to the 1:1 line (*y* = 0.56 + 0.76*x*, Figure 4A–2; *y* = −14.16 + 0.90*x*, Figure 4B–2). The fitting line of % K remaining deviated more from the 1:1 line (*y* = 3.88 − 0.03*x*, Figure 4A–4; *y* = 5.92 − 0.08*x*, Figure 4B–4).

Sediment burial affects the retention of different elements in *E. crusgali* and *B. tripartita* litters (Figure 3). The comparison of carbon loss and nutrient release characteristics of herbaceous plants of the same species at different burial depths showed that CNAI at 5 cm and 10 cm depth of *E. crusgali* and *B. tripartita* was greater than that at 0 cm depth at each decomposition time (30, 90, 180 d) (*p* < 0.05). The highest CNAI was 69.34% in the burial treatment and 56.38% in 0 cm burial depth. The remaining K efficiency at 10 cm depth (up to 1.71%) was significantly lower than at 0 cm depth (up to 7.10%). Interestingly, the P remaining in the litter in 5 cm and 10 cm buried depths of *E. crusgali* and *B. tripartita* at 180 d was significantly greater than at 30d (*p* < 0.05).

### 3.2. Change in Carbon and Nutrient Content in the Soil

**Figure 5:**
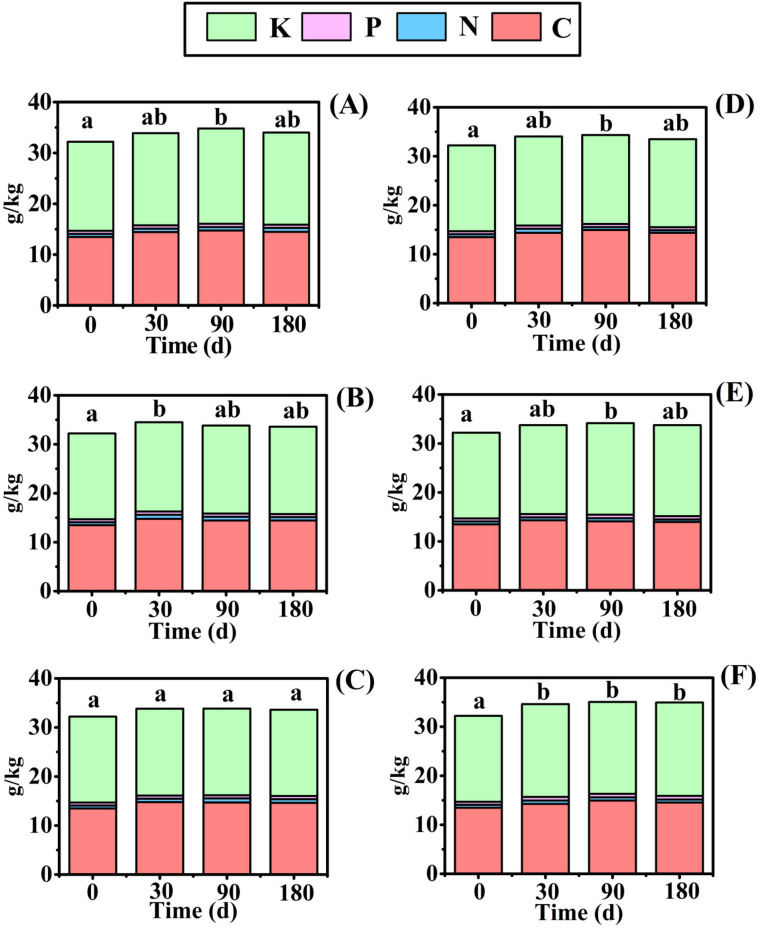
Total soil carbon, nitrogen, phosphorus, and potassium concentration of *E. crusgali* and *B. tripartita* litter decomposition after 0, 30, 90, 180 days at 0, 5, 10 cm burial depths under flooding. (**A**) *E. crusgali* at 0 cm; (**B**) *E. crusgali* at 5 cm; (**C**) *E. crusgali* at 10 cm; (**D**) *B. tripartita* at 0 cm; (**E**) *B. tripartita* at 5 cm; (**F**) *B. tripartita* at 10 cm. Different letters denote a significant difference in total soil nutrients among given dates (*p* < 0.05).

**Figure 6:**
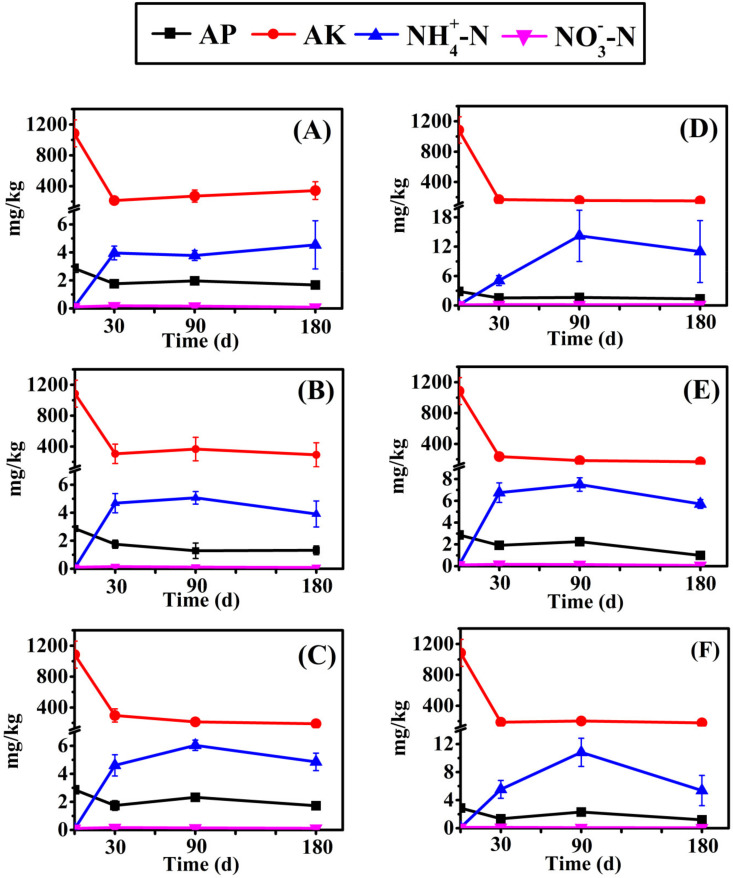
Soil available phosphorus, available potassium, ammonium nitrogen, and nitrate nitrogen concentration of *E. crusgali* and *B. tripartita* litter decomposition after 0, 30, 90, 180 days at 0, 5, 10 cm burial depths under flooding. (**A**) *E. crusgali* at 0 cm; (**B**) *E. crusgali* at 5 cm; (**C**) *E. crusgali* at 10 cm; (**D**) *B. tripartita* at 0 cm; (**E**) *B. tripartita* at 5 cm; (**F**) *B. tripartita* at 10 cm. Error bars indicate ± 1 S.D.

Soil carbon and nutrient content change in *E. crusgali* and *B. tripartita* litter decomposition in submergence is shown in Figure 5 and Figure 6. In the initial decomposition process, the total K content in the soil was the highest with the highest content up to 18.91 g/kg, then C up to 14.93 g/kg, and the N and P contents were not more than 1 g/kg. C and K content accounted for more than 95% of the total nutrient content and only a few N and P. Compared with the initial value, the total element contents in the 5 cm burial of *E. crusgali* (Figure 5B) and the 10 cm burial of *B. tripartita* (Figure 5F) were significantly increased at 30 d after decomposition (*p* < 0.05). Except for *E. crusgali* buried at 5 cm and 10 cm depths, total soil nutrient content increased significantly after 90 days of decomposition (*p* < 0.05). After 180 d, only the soil nutrient content at 10 cm depth of *B. tripartita* significantly increased compared with the initial value (*p* < 0.05). However, there was no significant change in the number of soil nutrients in all treatments in the decomposition process after 30 d. The soil available nutrient change was similar among different species and burial treatments. The soil’s initial AP and AK contents were 2.87 mg/kg and 1085.17 mg/kg, respectively. The soil AP and AK contents were significantly decreased at 30 d, and continuous change was not obvious after that. Inversely, N$H_{4}^{+}$–N initial content was 0.08 mg/kg, which increased significantly at 30 d, then reached 14.22 mg/kg at 90 d, and then decreased at 180 d. The N$O_{3}^{-}$–N content changed little in the decomposition process, and the content was not more than 0.2 mg/kg.

The differences in soil physicochemical properties were basically the same in each decomposition stage. The ORP and pH on the soil surface were higher than in the burial treatment, while the temperature was lower (Table 2). At 30 d and 90 d of decomposition, the ORP of burial treatment was negative and the lowest was −85.33 ± 45.39 mv, while the soil surface was positive and the highest was 468.67 ± 0.58 mv. At 180 d, ORP values were all positive, yet ORP under burial treatment was still greater than that without burial. At 90 days, the lowest temperature was around 7 °C, which was due to the seasonal climate. The soil pH value was up to 8.97 ± 0.05 at 90 d without burial and down to 6.06 ± 0.39 at 180 d under 5 cm burial.

### 3.3. Change in Nutrient Contents in the Overlying Water

**Figure 7:**
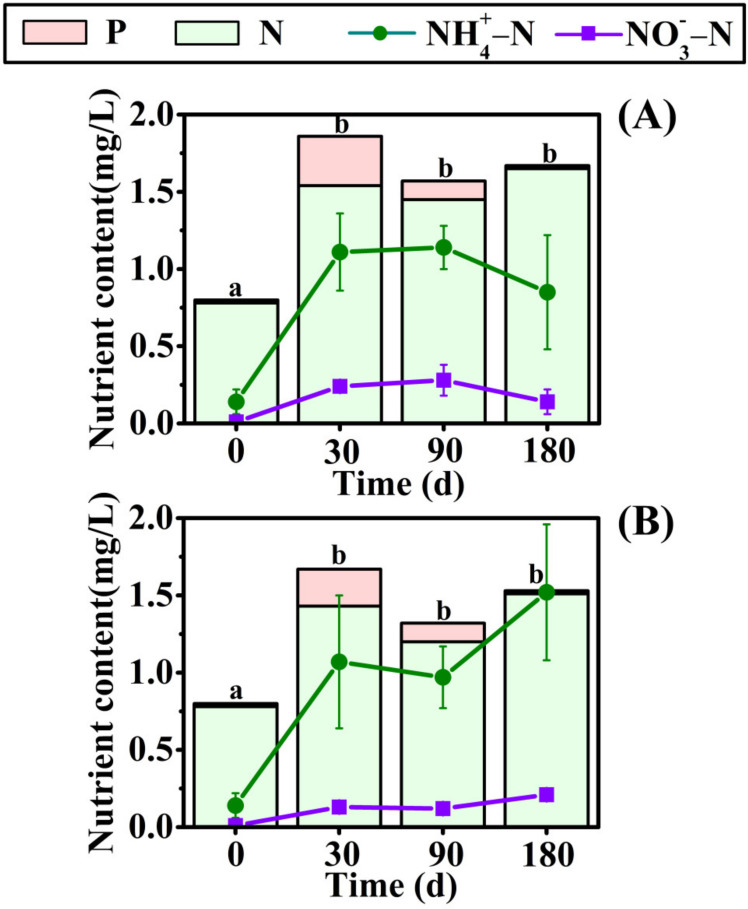
Change in nutrient content in overlying water in the decomposition of *E. crusgali* (**A**) and *B. tripartita* (**B**). Error bars indicate ± 1 S.E. Different letters denote a significant difference in total nitrogen and phosphorus in the water between given dates (*p* < 0.05).

The effects of the decomposition of *E. crusgali* (Figure 7A) and *B. tripartita* (Figure 7B) litter under waterlogging conditions on nutrient content in overlying water are shown in Figure 7. The initial contents of total nitrogen, phosphorus, ammonium, and nitrate nitrogen were 0.78 ± 0.17 mg/L, 0.02 ± 0.00 mg/L, 0.14 ± 0.08 mg/L, and 0.01 ± 0.00 mg/L, respectively. The total nitrogen and phosphorus, nitrate, and ammonium nitrogen content in the water increased significantly after 30 days of decomposition. However, the nutrient change in the water was not obvious after 30 days. This indicates that early litter decomposition strongly influences the nutrient content of the water.

### 3.4. Relationship between Nutrient Change and Soil Properties in Litter Decomposition

**Figure 8:**
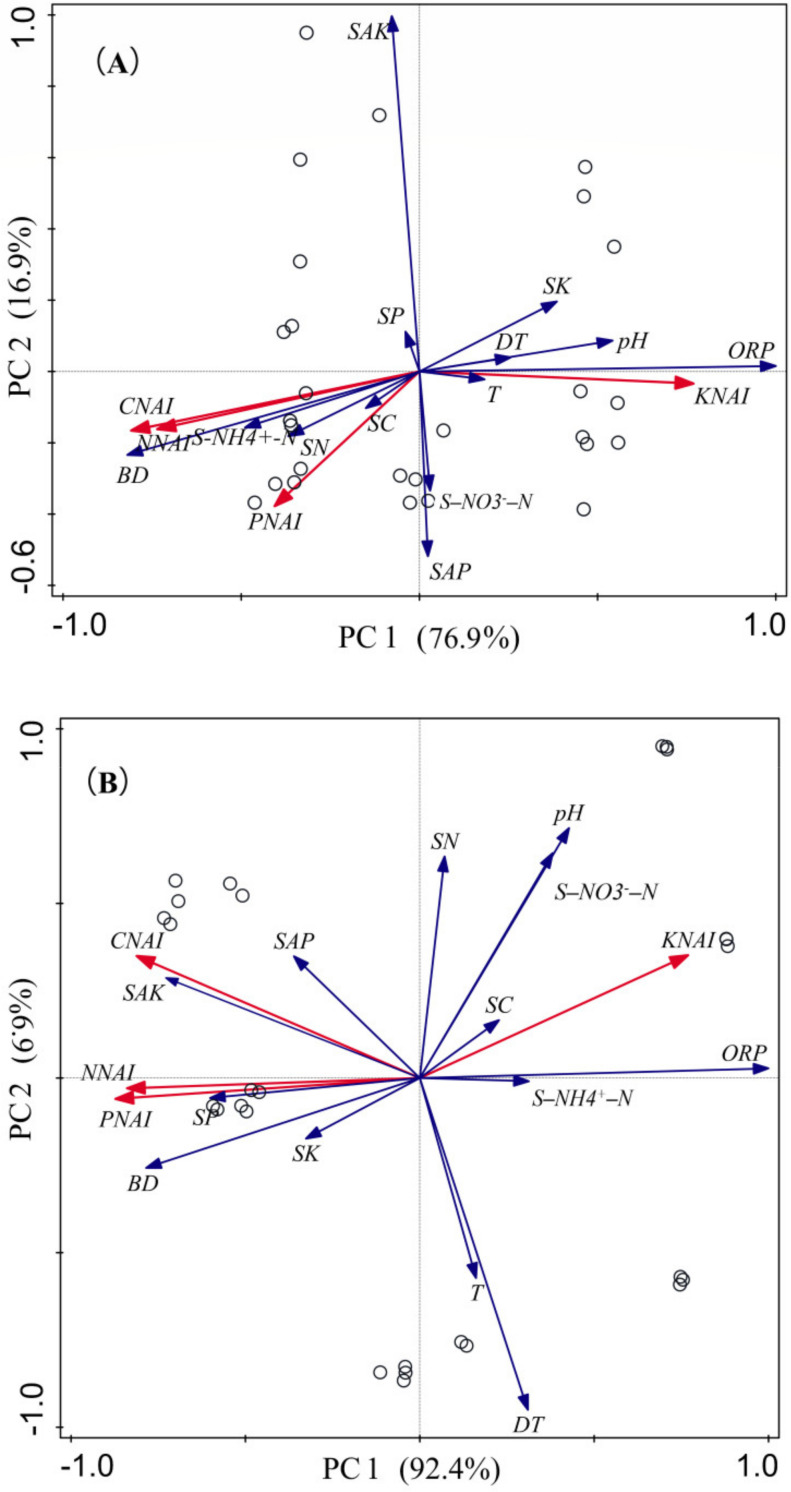
Principal component analysis (PCA) applied to data of *E. crusgali* (**A**) and *B. tripartita* (**B**) litter nutrient change during decomposition and different exoteric factors. CNAI: carbon NAI; NNAI: nitrogen NAI; PNAI: phosphorus NAI; KNAI: potassium NAI; BD: buried depth; DT: decomposition time; ORP: soil oxidation–reduction potential; T: soil temperature; pH: soil pH; SC: total soil carbon; SN: total soil nitrogen; SP: total soil phosphorus; SK: total soil potassium; SAP: soil available phosphorus; SAK: soil available potassium; S–NH4^+^–N: soil ammonium nitrogen; S–NO3^−^–N: soil nitrate nitrogen.

Principal component analysis (PCA) was used to assess the overall effect of various environmental variables on litter nutrient release, including soil nutrient content and physicochemical properties. The PCA results of litter decomposition and nutrient release of *E. crusgali* and *B. tripartita* are shown in Figure 8. The main axis 1 in Figure 8A,B has the highest explanatory degree of soil redox potential ORP in PCA and is understood as an exoteric factor.

As shown in Figure 8A,B, the accumulation coefficient vectors of litter C, N, and P showed a positive correlation (acute angles between the vectors). At the same time, the three nutrient values were negatively correlated with the K accumulation of litter. Furthermore, the litter’s C, N, and PNAI were significantly positively correlated with burial depth but negatively correlated with soil redox potential ORP. On the contrary, KNAI was negatively correlated with buried depth and significantly positively correlated with ORP. This indicates that the ORP decreases as soil depth increases. Interestingly, the nutrient change in *E. crusgali* litter was observed to correlate with decomposition time, while other elements of *B. tripartita*, except C, had no strong correlation with time.

## 4. Discussion

The existence forms of different nutrient elements in litter may be different, resulting in different release rates. As shown in Figure 3 and Figure 4, KNAI is the lowest of all burial depth treatments of *E. crusgali* and *B. tripartita*. That is, before 30 days of litter decomposition, compared to C and nutrient elements, the K release rate was the largest, and the KNAI change was insignificant after 30 days, which is consistent with previous research results [32,33,34,35]. Litter decomposition in aquatic environments mainly includes leaching and microbial degradation, followed by mechanical and invertebrate fragmentation [36]. Leaching is the removal of substances from plants by the action of water. Different elements can be leached from plants to varying degrees, mostly determined by the present forms of elements in plants. In the first weeks of decomposition, the litter’s soluble organic and inorganic substances can be rapidly lost into the environment mainly by the scouring and leaching of water bodies and microbial degradation, resulting in rapid litter mass consumption [15,37]. At the latter stage of decomposition, some stubborn substances in the litter are slowly mineralized mainly under the action of microbial decomposers, resulting in the gradual loss of C and some nutrient elements. K is the most easily mobile nutrient element in the litter. The K element exists in soluble compounds in the form of ions and can cause rapid erosion [33,34]. Furthermore, the K element of litter is easily preferentially consumed by microorganisms [9,38], so the K element is released most compared to other nutrient elements in early decomposition, and there is no change over time [39].

In this study, the element loss in the decomposition process followed K > P > N > C based on a comparison of different nutrient elements in litter. This outcome is contrary to that of Robert (2010), and a possible explanation for this might be C/N [34]. Nitrogen mineralization generally occurs between 20:1 and 30:1 C/N, and when C/N is above this level, nitrogen will be fixed by microorganisms on litter [40]. In this study, the initial C/N ratio in litter is greater than 30:1 (Table 1), so the N element release is relatively slow. Our results corroborate the findings of Edmonds (2010) that the C element retention rate is always the highest in the decomposition process [34]. The main factor is that C exists in plants in energy storage substances such as soluble sugars and lipids and supporting substances such as cellulose, hemicellulose, lignin, and phenols [41]. As shown in Figure 4, the relationship between the % C remaining and the % mass remaining was nearly 1:1, indicating that C constitutes structural components in the plant. In later decomposition, based on the reduction of soluble organic matter and other readily decomposable substances, lignin and other recalcitrant substances were gradually retained and slowly decomposed by microorganisms [42]. N and P are the nutrient origins of decomposer microorganisms [43], and N:P in microorganisms is 12:1. Hence, the P element is a controlling factor for microbial growth [13]. Because of the large absorption degree of P by microorganisms and the lower P content to N in the litter, the residual rate of P is lower than that of N. The N increase in litter over decomposition has been shown in studies, which is due to the combination of exogenous N with microbial biomass [44].

However, this study did not find that one reason may be the reduced activity of microorganisms in water and low temperatures, resulting in less nitrogen enrichment in litter microorganisms. P presents a state of leach–enrich release throughout the experiment [45], which is related to the nutrient absorption of microorganisms [46]. Studies have shown that P is a revolving element in the growth of microorganisms, and when microorganisms grow to a certain stage, they will re-release P [39,47,48]. Environmental change buried in sediment may also be one reason for the release of P by the microorganism, and further studies are needed in this respect.

Decomposition releases nutrients from litter depending on the surrounding environment. Some nutrients are returned to the soil and re-absorbed by plants, thus being transferred to other organisms in the food chain [49]. The soil carbon and nutrient content change in the litter decay process is shown in Figure 5 and Figure 6. The soil’s total K and C contents are relatively high, while the initial N and P values are low, and they are microbial growth materials, so the content change is not obvious. The soil AP and AK content decreased significantly in the early stage of litter decomposition. One explanation is that colonization and growth need microorganisms before decomposition and leaching into the water. In addition, microbial decomposition ability may always be inhibited by underwater flooding conditions [19]. Soil nutrients may be transferred into the water environment, resulting in reduced nutrients (Figure 7) [50].

Following the results of Niu (2019), at a depth of 0 cm for the two herbaceous plants, the soil’s total nutrient content did not significantly increase within 30 days of decay [20]. In the early stage, the litter decomposition products were mainly highly soluble and low-molecular-weight organic acids, which could not be retained in the soil, resulting in a significant increase in the nutrient content of the overlying water. The soil nutrient content increased significantly when the decomposition time reached 90 days, indicating that the nutrient input at 90 days was greater than the nutrients lost from the decomposition process, increasing soil nutrient content. One reasonable explanation is that late in the decomposition process, in an experiment conducted in the winter, a decrease in temperature resulted in a decrease in microorganism activity. The litter left behind is composed of tenacious materials such as cellulose [9]. These slow the efficiency of litter nutrient loss [51], but soil nutrients migrate to the water, decreasing soil nutrient content. Moreover, the migration rate of surface soil nutrients to the water may be faster than the deeper soil. Studies have shown that litter decomposition will slow down under sediment burial, decreasing nutrient release efficiency [52,53]. The pH and ORP in the surface soil were higher than in the deep soil, and the temperature was lower in Table 2. This may alter the activity of the decomposing microorganism as a possible cause of the different nutrient release rates, so the soil nutrient increase was not obvious under 5 cm of soil.

In addition to the migration of soil nutrients to the water, other factors such as microorganisms also strongly affect soil nutrient migration. For example, soil microbial denitrification can cause soil N elements in the form of inorganic substances to be released into the atmospheric environment [13]. Still, the aquatic fungus nutrient retention rate is lower than for terrestrial plants [15]. In addition, litter microorganisms breathing under flooding emit greenhouse gas emissions of CO_2_, CH_4_, and N_2_O into the atmosphere during the decomposition process [29,54,55], resulting in an imbalance between the increased intensity of soil and the decrease in litter nutrients. Simultaneously, previous studies have shown that plant litter traits are important decomposition factors, and the litter of species with tough leaves is harder to decompose than soft leaves [56,57]. *E. crusgali* and *B. tripartita*, respectively, belong to *Poaceae* and *Asteraceae*, with different growth characteristics. The slender leaves and soft stems of *E. crusgali* and hard stalks of *B. tripartita* lead to *Poaceae* litter’s rapid rate of decomposition under the same conditions. However, under the *B. tripartita* 10 cm depth, soil nutrients increased significantly. This may be attributed to the higher nutrient concentration and the reductive environment underwater [8,58].

The results presented in Figure 8 showed that redox potential is negatively correlated with soil burial depth. Redox potential decreases as the soil is buried, and reducing conditions promote soil nutrient decrease [59]. Moreover, studies have shown that anaerobic microorganisms can produce organic acids under waterlogged conditions, resulting in soil pH declining [60,61,62], yet the activity of microorganisms is weakened in a low pH soil environment [8], leading to a decline in their feeding capacity for C, N, and P and a slower decomposition rate. Simultaneously, the litter decomposition buried by sediment is inhibited, so the residual amount of C, N, and P of litter buried under the soil is higher than the surface soil. Yet, K is found in higher concentrations in soluble substances. Microorganisms in deeper soil settle more densely on litter during the early stages of decomposition [63], resulting in a rapid loss [64]. Consequently, KNAI has an inverse relationship with burial depth. In addition, the stubborn litter of *B. tripartita* and the microorganisms continuing to absorb external nutrients during the decomposition process resulted in poised elements except for C as decomposition progressed [36].

## 5. Conclusions

The litter decomposition rate over the 180 d sediment burial under flooding conditions was initially rapid, but subsequently became sluggish. The nutrient loss rate was K > P > N > C. The relationship between % C remaining and % mass remaining was nearly 1:1, while the K content did not change significantly after the rapid release in the early stage and the total amount of P presented a leach–release–enrichment state in the decomposition process, which was mainly related to the existing forms of elements and microbial activities. In addition, the nutrients in the soil and overlying water increased significantly at the first decomposition stage. Still, the total soil nutrients changed insignificantly at the end, except for the 10 cm burial of *B. tripartitle*. This is related to water leaching and the transformation of soil nutrients. Sediment burial affects litter mass and carbon loss and nutrient release, mainly due to ORP differences at different soil depths. The surrounding environmental differences contribute to changes in the microorganisms and decomposers’ activity, resulting in differences in decomposition. This study shows that sediment inhibited the C loss and release of N and P elements but stimulated the release of K element. The litter decomposition of annual herbaceous plants has little effect on the total soil nutrients under waterlogging conditions. Nonetheless, litter does more to the soil than that. It is essential to investigate the continuing production of residual soil litter nutrients after the water level has receded. This study helps understand the dynamic characteristics of herbaceous plant nutrients during decomposition and their effects on the material circulation of the ecosystem. This study provides useful information for plant growth and pollution remediation in the water fluctuation zone.

## Figures and Tables

**Table 1:** Initial value of nutrient contents and their ratios in the litter of *Echinochloa crusgali* and *Bidens tripartita*.

Species	g C kg^−1^	g N kg^−1^	g P kg^−1^	g K kg^−1^	C/N	C/P
*Echinochloa crusgali*	384.17 ± 2.16	12.20 ± 0.53	3.56 ± 0.21	16.73 ± 1.05	31.53 ± 1.51	108.24 ± 6.77
*Bidens tripartita*	428.70 ± 0.87	12.07 ± 0.35	3.87 ± 0.14	14.5 ± 1.06	35.55 ± 0.97	110.91 ± 3.97

Note: Data are means ± S.E.

**Table 2:** Soil oxidation–reduction potential, temperature, and pH values of *E. crusgali* and *B. tripartite* litter after 30, 90, and 180 days at 0, 5, 10 cm burial depths under flooding.

Species	Time	Buried Depth	ORP/mv	T/°C	pH
*E. crusgali*	30 d	0 cm	416.33 ± 1.15 a	13.23 ± 0.12 b	8.37 ± 0.06 a
		5 cm	−34.67 ± 11.59 b	14.20 ± 0.36 a	7.79 ± 0.18 b
		10 cm	−52.33 ± 23.46 b	14.17 ± 0.12 a	7.73 ± 0.03 b
	90 d	0 cm	468.67 ± 0.58 a	7.03 ± 0.06 a	8.97 ± 0.05 a
		5 cm	−48.33 ± 24.01 b	7.03 ± 0.12 a	7.5 ± 0.06 b
		10 cm	−71.67 ± 39.88 b	7.23 ± 0.42 a	7.53 ± 0.05 b
	180 d	0 cm	413.33 ± 2.52 a	20.03 ± 0.12 b	8.08 ± 0.07 a
		5 cm	141.33 ± 48.95 b	20.60 ± 0.30 a	6.79 ± 0.15 b
		10 cm	124.33 ± 12.58 b	20.33 ± 0.12 ab	6.47 ± 0.39 b
*B. tripartite*	30 d	0 cm	407.67 ± 2.31 a	13.30 ± 0.10 b	8.36 ± 0.21 a
		5 cm	−78.67 ± 30.89 b	14.13 ± 0.06 a	7.75 ± 0.10 b
		10 cm	−85.33 ± 45.39 b	14.30 ± 0.10 a	7.87 ± 0.07 b
	90 d	0 cm	465.00 ± 0.00 a	7.03 ± 0.06 a	8.94 ± 0.12 a
		5 cm	−32.00 ± 10.58 b	7.00 ± 0.10 a	7.42 ± 0.06 b
		10 cm	−52.67 ± 22.28 b	7.03 ± 0.15 a	7.44 ± 0.10 b
	180 d	0 cm	408.00 ± 2.00 a	20.17 ± 0.15 b	8.15 ± 0.06 a
		5 cm	113.00 ± 13.08 b	21.47 ± 0.29 a	6.06 ± 0.39 b
		10 cm	164.33 ± 35.85 b	20.97 ± 0.31 a	6.41 ± 0.47 b

Note: ORP: oxidation–reduction potential; T: temperature; means ± S.D. Different letters denote a significant difference in soil properties among given buried depths (*p* < 0.05).

## Data Availability

The data presented in this study are available in the figures and tables.

## Supplementary Materials

The following are available online at https://www.mdpi.com/article/10.3390/biology10111141/s1, Table S1: Results of repeated measure ANOVA for nutrient NAI values of *E. crusgali* and *B. tripartite*.
