# Supplementary material for: Response of Annual Herbaceous Plant Leaching and Decomposition to Periodic Submergence in Mega-Reservoirs: Changes in Litter Nutrients and Soil Properties for Restoration
Source: Biology (Basel). 2021 Nov 5;10(11):1141. doi: 10.3390/biology10111141 (PMC8614921; doi:10.3390/biology10111141)
Supplement: Supplementary file 1 [file biology-10-01141-s001.zip › biology-1413931-supplementary.pdf]

Table S1. Results of repeated measure ANOVA for nutrient NAI values of *E. crusgali* and *B. tripartite*

Source of variation	*F* value			
	**C**	**N**	**P**	**K**
Species	82.921***	13.16**	88.949***	106.364***
Buried depth	554.835***	133.052***	134.505***	299.708***
Time	491.816***	23.617***	3.421*	34.691***
Species × Buried depth	1.002	0.958	45.415***	9.183**
Species × Time	22.152***	3.825*	9.716***	5.966**
Buried depth × Time	15.075***	3.147*	6.046**	3.925*
Species × Buried depth × Time	1.197	0.550	5.51**	7.677***

Note**:** C: total carbon NAI; N: total nitrogen NAI; P: total phosphorus NAI; K: total potassium NAI. *: P<0.05; **: P<0.01; ***: P<0.001.
